# Initial assessment of the COVID-19 vaccination’s impact on case numbers, hospitalisations and deaths in people aged 80 years and older, 15 EU/EEA countries, December 2020 to May 2021

**DOI:** 10.2807/1560-7917.ES.2021.26.48.2101030

**Published:** 2021-12-02

**Authors:** Nathalie Nicolay, Francesco Innocenti, Julien Beauté, Veronika Učakar, Marta Grgič Vitek, Eero Poukka, Tuula Hannila-Handelberg, Charmaine Gauci, Tanya Melillo, Theano Georgakopoulou, Jiri Jarkovsky, Pavel Slezak, Concepción Delgado-Sanz, Carmen Olmedo-Lucerón, Heleene Suija, Rasa Liausediene, Piaras O’Lorcain, Niamh Murphy, André Peralta-Santos, Pedro Casaca, Ioanna Gregoriou, Nick Bundle, Gianfranco Spiteri, Giovanni Ravasi

**Affiliations:** 1 European Centre for Disease Prevention and Control (ECDC), Solna, Sweden; 2 Epidemiology Unit, Regional Health Agency of Tuscany, Florence, Italy; 3 National institute of Public Health, Ljubljana, Slovenia; 4 Finnish Institute for Health and Welfare, Helsinki, Finland; 5 Health Promotion and Disease Prevention Directorate, Msida, Malta; 6 Department of Epidemiological Surveillance and Intervention of the National Public Health Organization (NPHO), Athens, Greece; 7 Data analysis department, Institute of Health Information and Statistics of the Czech Republic, Prague, Czechia; 8 Department of Infectious Diseases Epidemiology, National Institute of Public Health, Prague, Czechia; 9 National Epidemiology Centre, CIBERESP, Institute of Health Carlos III, Madrid, Spain; 10 Area Immunisation Programme. Ministry of Health, Madrid, Spain; 11 Department of Communicable Diseases, Health Board, Tallin, Estonia; 12 National Public Health Centre, Vilnius, Lithuania; 13 Health Protection Surveillance Centre, Dublin, Ireland; 14 Directorate of Information and Analysis, Directorate-General of Health, Lisbon, Portugal; 15 Ministry of Health, Nicosia, Cyprus

**Keywords:** COVID-19, vaccination uptake, mass vaccination, health impact assessment

## Abstract

Prioritisation of elderly people in COVID-19 vaccination campaigns aimed at reducing severe outcomes in this group. Using EU/EEA surveillance and vaccination uptake, we estimated the risk ratio of case, hospitalisation and death notifications in people 80 years and older compared with 25–59-year-olds. Highest impact was observed for full vaccination uptake 80% or higher with reductions in notification rates of cases up to 65% (IRR: 0.35; 95% CI: 0.13–0.99), hospitalisations up to 78% (IRR: 0.22; 95% CI: 0.13–0.37) and deaths up to 84% (IRR: 0.16; 95% CI: 0.13–0.20).

The rollout of vaccines against coronavirus disease (COVID-19) started between late December 2020 and mid-January 2021 in all 30 European Union and European Economic Area (EU/EEA) countries [1]. By May 2021, the Comirnaty (BNT162b2 mRNA, BioNTech-Pfizer, Mainz, Germany/New York, United States (US)), Spikevax (mRNA-1273, Moderna, Cambridge, US), Vaxzevria (ChAdOx1 nCoV-19, Oxford-AstraZeneca, Cambridge, United Kingdom), and COVID-19 Vaccine Janssen (Ad26.COV 2.5, Janssen-Cilag International NV, Beerse, Belgium) vaccines had received approval from the European Medicine Agency (EMA). Because of limited vaccine supplies, most EU/EEA countries prioritised healthcare workers (HCWs), individuals aged 80 years and older and residents in long-term care facilities (LTCF) in the initial phase of the vaccination rollout [2]. We aimed to assess the impact of COVID-19 vaccination on notification rates of COVID-19 cases, related hospitalisations and deaths in those 80 years and older, using routine surveillance data collected by the European Centre for Disease Prevention and Control (ECDC).

## Surveillance of COVID-19 cases and vaccination

Since 27 January 2020, ECDC and the World Health Organization (WHO) Regional Office for Europe coordinate COVID-19 surveillance in Europe [3]. All EU/EEA countries report on a weekly basis either case-based or aggregated COVID-19 data to the European Surveillance System (TESSy) using the EU case definition [4]. Variables collected in the case-based dataset include main demographic data, COVID-19-related hospitalisation and/or intensive care unit (ICU) admission and outcome (alive, death -related or not- to COVID-19 or, still in treatment). In addition, the ECDC epidemic intelligence team collects daily the number of COVID-19 cases and deaths reported for EU/EEA countries by official sources such as Ministries of Health or National Public Health Institutes [5].

Since 15 January 2021, as part of a joint ECDC/WHO surveillance, EU/EEA countries report to TESSy weekly aggregate data on the number of administered COVID-19 vaccine doses by vaccine product and age group (all, 18–24, 25–49, 50–59, 60–69, 70–79 years and 80 years and older), as well as in HCWs and LTCF residents [6]. The vaccination uptake for at least one dose is defined as the number of first doses administered divided by the size of the total population of a specific target group. For full vaccination uptake the number of persons fully vaccinated as per manufacturers’ instruction is used as the numerator.

## Modelling the impact of COVID-19 vaccination

Using Poisson regression, we assessed the impact of vaccination by modelling the ratio of the rate of weekly notifications of COVID-19 cases, hospitalisations and deaths in those aged 80 years and older and in 25–59-year-olds (reference group) from week 48 2020 to week 20 2021. We assumed that within an age group, case detection and notification has been stable over the study period for a specific country and that variations in the ratio were mostly driven by the implementation of the vaccination. The ratio allowed us to control to some extent, for changes in the epidemics, assuming that the ratio would not be much affected by epidemic trends in the absence of age-specific interventions. During the early phase of the vaccine rollout, younger age groups were not prioritised (with the exception of specific vulnerable individuals or occupational risk groups). Therefore, the ratio between the rates for the above listed outcomes in those 80 years and older over the 25–59-year-olds reflects the impact of the vaccination when assessed alongside uptake, to a large extent.

For each outcome, we fitted models adjusting for cumulative vaccine uptake for ‘at least one dose’ and separately for ‘full vaccination’ in those 80 years and older, stratified in five uptake groups (< 20%, 20–39%, 40–59%, 60–79%, ≥ 80%). All models were adjusted for reporting country. Results are presented as incidence rate ratios (IRRs) with 95% confidence intervals (CI). All analyses were performed using Stata 16.0 (StataCorp. 2019. College Station, Texas, US: StataCorp LLC).

For numbers of cases and deaths, we included countries reporting at least 80% to TESSy compared with the figures collected by the ECDC epidemic intelligence team [7]. No completeness threshold was applied for countries reporting on hospitalisations in the case-based data set (Table 1). We ran sensitivity analyses applying thresholds of 50% and 60% completeness for the hospitalisation variable and the results are in line with our main analysis. (Supplementary material). Since vaccination does not provide immediate protection, we allowed delayed exposure effects based on available evidence. Thus, we used a two-week lag for case notification, hospitalisation, and death rates [8,9].

**Table 1:** Summary of TESSy data completeness and hospitalisation variable completeness, 15 EU/EEA countries as at 16 June 2021

Country	Completeness^a^ of data reported to TESSy, to week 23/2021 (%)		Variable completeness^b^ in TESSy, weeks 48/2020 to 20/2021 (%)
	Cases	Deaths	Hospitalisation
Austria	99.7	99.8	56.6
Cyprus	97.9	122	100.0
Czechia	100	99.9	73.2
Estonia	98.0	98.4	No data available
Finland	98.2	125.1	65.6
Greece	98.9	100.2	No data available
Ireland	94.9	94.1	77.2
Italy	99.5	99.8	100.0
Latvia	98.5	111.4	No data available
Lithuania	95.2	85.2	No data available
Malta	100	90.7	100.0
Portugal	100	100	1.6
Slovenia	100	100	No data available
Spain	99.9	100	No data available
Sweden	98.0	98.5	2.1

ECDC: European Center for Disease Prevention and Control; EU/EEA: European Union and European Economic Area; TESSy: The European Surveillance System.

^a^ Proportion of case-based reports to TESSy compared with number of cases collected by ECDC’s epidemic intelligence from official sources (Ministries of Health or National Public Health Institutes).

^b^ Proportion of records in TESSy with known status.

## Impact of COVID-19 vaccination on rates of notified cases, hospitalisations and deaths

Nine countries were included in the analysis of hospitalisation rates (Austria, Cyprus, Czechia, Finland, Ireland, Italy, Malta, Portugal, and Sweden) and six additional countries in the analysis of case and death notifications rates (Estonia, Greece, Latvia, Lithuania, Slovenia and Spain). All countries had started the vaccination rollout by week 01 2021. Non-EU countries do not report case-based data nor uptake by detailed age group and were not part of this analysis.

**Figure 1:**
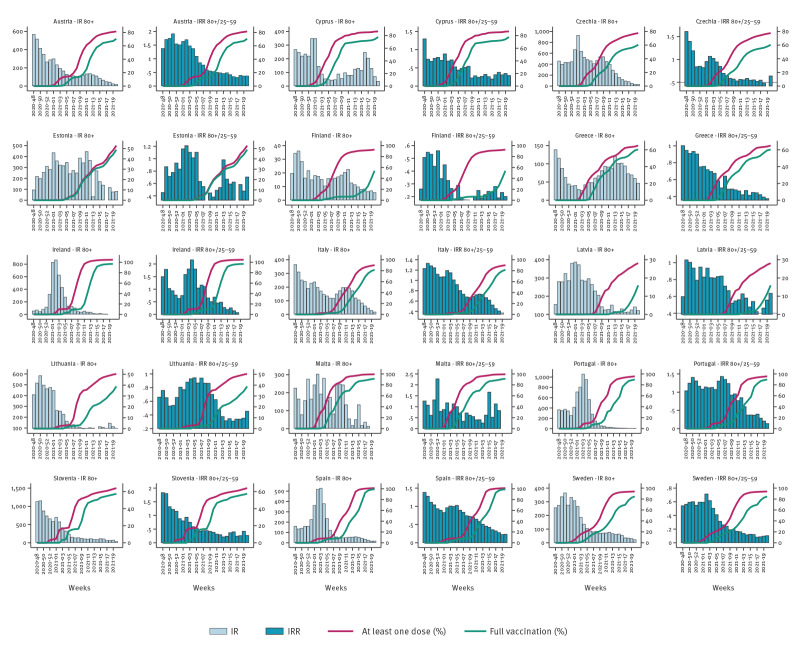
Incidence rate for case notifications in those 80 years and older, incidence rate ratio in those 80 years and older versus in 25–59-year-olds, and vaccine uptake of at least one dose or full vaccination in those 80 years and older by country, 15 EU/EEA countries, week 48 2020−week 20 2021

Trends and magnitude of weekly case notification rates in those aged 80 years and older differed across participating countries over the study period (Figure 1) with some countries having decreasing trends and other countries having no clear overall pattern. Peak values were above 300 per 100,000 population in most countries except in Greece (< 150), Finland and Norway (< 50/100,000). By week 20 2021, in people aged 80 years and older, the vaccine uptake for at least one dose ranged from 32.2% in Latvia to 100% in Ireland, Malta, Spain, and full vaccination uptake between 19.1% in Latvia and 100% in Ireland. The level of uptake in 18–59-year-olds was considerably lower as the rollout for this age group was delayed (Table 2).

EU/EEA: European Union and European Economic Area.

For each country, the first panel label for the left axis (y-axis) is ‘Incidence rate in those 80 years and older per 100,000 population’ and the label for the axis on the right is ‘Vaccine uptake (%). In the second panel for each country, the left axis (y-axis) label is ‘Incidence rate ratio of those 80 years and older versus 25−59 year-olds’ and the label for the right axis is ‘Vaccine uptake (%).

**Table 2:** COVID-19 vaccine uptake with either at least one dose or full vaccination, 15 EU/EEA countries as at week 20 2021

Country	25–59-year-olds		80 years and older	
	At least one dose	Full vaccination	At least one dose	Full vaccination
Austria	35.3	9.8	81.5	70.5
Cyprus	43.0	15.3	86.7	77.8
Czechia	27.9	7.4	77.5	60.5
Estonia	22.2	12.3	53	48.9
Finland	34.7	4.5	92.6	53.9
Greece	23.1	7.6	65.0	60.4
Ireland	31.9	8.3	100.0	96.9
Italy	22.5	10.8	90.1	81.7
Latvia	19.7	6.4	28.1	16.0
Lithuania	32.1	13.7	50.3	38.9
Malta	57.4	29.7	99.4	91.2
Portugal	18.0	9.4	95.7	89.9
Slovenia	24.6	6.8	64.1	57.4
Spain	21.6	6.3	100.0	99.2
Sweden	24.9	5.6	93.9	84.7

COVID-19: coronavirus disease; ECDC: European Center for Disease Prevention and Control; EU/EEA: European Union and European Economic Area.

Source: ECDC Vaccine Tracker (https://vaccinetracker.ecdc.europa.eu/public/extensions/COVID-19/vaccine-tracker.html#uptake-tab).

**Figure 2:**
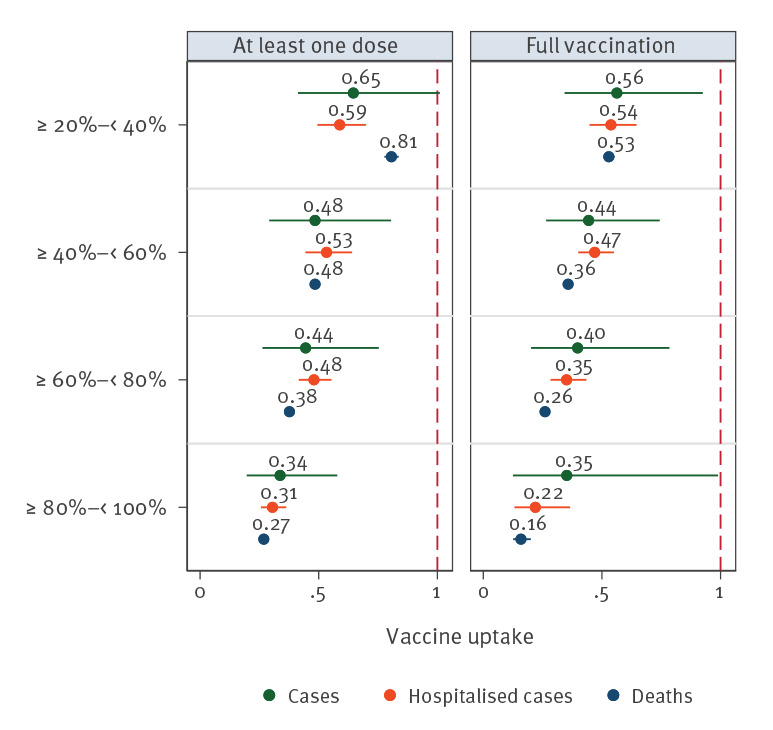
Adjusted incidence rate ratios for those 80 years and older versus 25–59-year-olds for case, hospitalisation and death notification rates, 15 EU/EEA countries^a^, week 48 2020−week 20 2021

In the model adjusted for reporting country, the ratio between the case notification rates among 80 year olds and older and in the 25–29-year-olds, compared with countries with vaccine uptake of below 20%, decreased by between 44% (IRR: 0.56; 95% CI: 0.34–0.93) among countries with full vaccine uptake of 20–39% and 65% (IRR: 0.35; 95%CI: 0.13–0.99) among countries with > 80% full vaccine uptake (Figure 1 and 2, Supplementary Table 1).

EU/EEA: European Union and European Economic Area.

^a ^Countries included: Austria, Cyprus, Czechia, Estonia, Finland, Greece, Ireland, Italy, Latvia, Lithuania, Malta, Portugal, Slovenia, Spain, Sweden in the case and hospitalisation notifications analysis and 9 countries included: Austria, Cyprus, Czechia, Finland, Ireland, Italy, Malta, Portugal, Sweden in the death notifications analysis.

For each country, the first panel label for the left axis (y-axis) is ‘Incidence rate in those 80 years and older per 100,000 population’ and the label for the axis on the right is ‘Vaccine uptake (%). In the second panel for each country, the left axis (y-axis) label is ‘Incidence rate ratio of those 80 years and older versus 25−59 year-olds’ and the label for the right axis is ‘Vaccine uptake (%).

**Figure 3:**
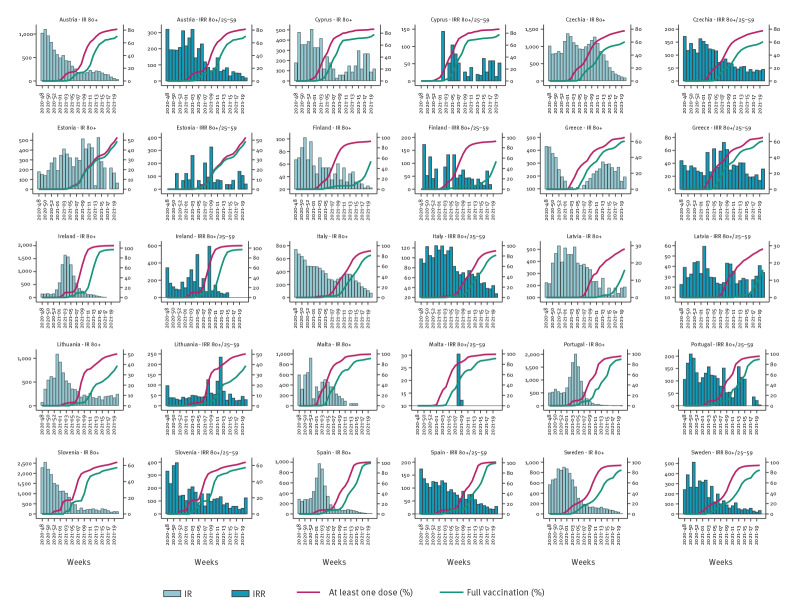
Incidence rate for hospitalisations in those 80 years and older, incidence rate ratio of hospitalisations in those 80 years and older versus 25–59-year-olds, and vaccine uptake of at least one dose or full vaccination in those 80 years and older by country, 15 EU/EEA countries, week 48 2020 to week 2 2021

**Figure 4:**
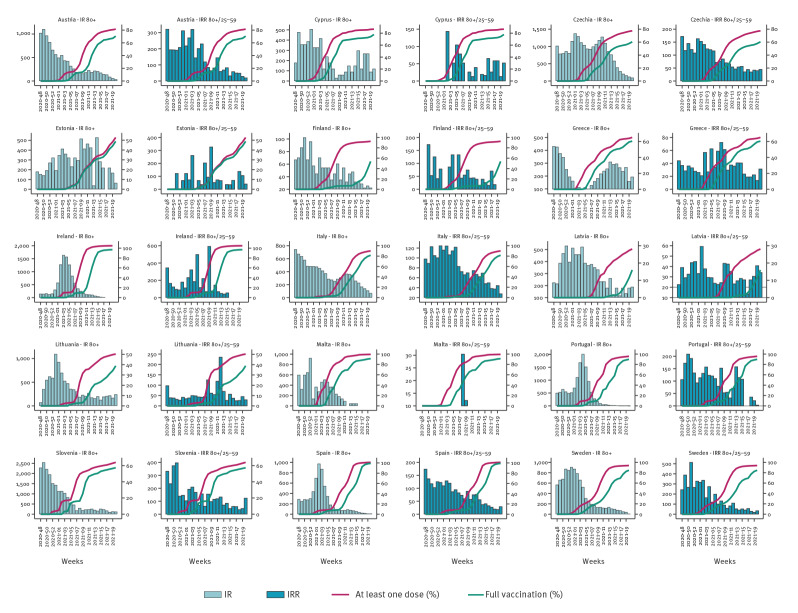
Incidence rate for deaths in those 80 years and older, incidence rate ratio of deaths in those 80 years and older versus 25–59-year-olds, and vaccine uptake of at least one dose or full vaccination in those 80 years and older by country, 15 EU/EEA countries; week 48 2020 to week 20 2021

Similarly, the rate ratio varied from 46% (IRR: 0.54; 95% CI: 0.45–0.65) to 78% (IRR: 0.22; 95%CI: 0.13–0.37) for hospitalisation notifications (Figure 2 and 3, Supplementary Tables 2, 3, 4) and from 47% (IRR: 0.53; 95%CI: 0.51–0.55) to 84% (IRR: 0.16; 95% CI 0.13–0.20) for death notifications (Figure 2 and 4, Supplementary Table 5). The lowest IRR was found for death notifications when the level of vaccine uptake was at 80% or higher.

EU/EEA: European Union and European Economic Area.

EU/EEA: European Union and European Economic Area.

For each country, the first panel label for the left axis (y-axis) is ‘Incidence rate in those 80 years and older per 100,000 population’ and the label for the axis on the right is ‘Vaccine uptake (%). In the second panel for each country, the left axis (y-axis) label is ‘Incidence rate ratio of those 80 years and older versus 25−59 year-olds’ and the label for the right axis is ‘Vaccine uptake (%).

## Discussion

The primary objective of the vaccination campaigns in Europe was to reduce the burden of hospitalisation and death associated with SARS-CoV-2 (severe acute respiratory syndrome coronavirus 2) infections [10].

Our results indicated that among individuals aged 80 years and older in the included countries, the increase in full vaccination uptake coincided with a marked decline in hospitalisations and death notifications. These results show that full vaccination of the elderly population can be an effective public health measure to mitigate the impact of the pandemic on these severe outcomes. Vaccination also impacted on the overall case notification in those aged 80 years and older, albeit with wide CI.

This initial assessment covers the early phase of the European vaccination campaigns up to June 2021, before countries started considering to recommend an additional dose of the COVID-19 vaccine for possible waning immunity and reduced effectiveness against the SARS-CoV-2 Delta variant. There is now evidence that effectiveness of the vaccines may be reduced against Delta-variant infection but it remains high against severe outcomes [11].

The high IRR estimates for at least one dose may reflect the quick completion of the full vaccine course among elderly people in the early phase of the campaigns. Among individuals who had received at least one dose, some had completed the full recommended vaccination scheme. Therefore, our results should not be taken as evidence to encourage deferral of second dose to a later time point. Reports showed higher vaccine effectiveness on various outcomes after the completion of the full vaccination course [9]. However, it will be important to undertake an analysis of the impact of the vaccination campaigns across countries that implemented different strategies, in particular those who prioritised a broad application of the first dose over a two-dose vaccination schedule [12].

The analysis has several limitations. First, this is an ecological study and we did not have access to individuals’ vaccination statuses nor their disease outcome records that would have permitted us to assess the impact prospectively as well as the specific impact of partial vaccination. Second, the impact of vaccination may have been overestimated in the initially prioritised fragile population [13]. Among elderly people, the impact on those living in LTCFs needs to be assessed separately. Moreover, case detection and healthcare seeking behaviours may differ when comparing the elderly population with the general population.

Ongoing monitoring of the impact of vaccination is required as it may be influenced by the lifting of non-pharmaceutical interventions (or their future reinstallation), the emergence of more transmissible variants [14], and a possible waning immunity over time, following the second dose [15].

### Conclusion

We show that vaccination has proved to be an effective measure to prevent severe outcomes in those aged 80 years and older. Reduction of disease burden in the elderly population was achieved with the rapid deployment of the vaccine whether or not, other public health interventions remained in place in the countries over spring 2021. Further analysis is required to assess (i) the impact of possible waning immunity in this vulnerable population group, (ii) the circulation of the Delta or other emerging variants and (iii) the lifting of non-pharmaceutical interventions. The impact of administering an additional vaccine dose for older people which is now widely recommended in Europe, could be assessed using a similar methodology as the one described in this paper.

## Supplementary Data

475000 Supplementary Material
