# Supplementary material for: Initial assessment of the COVID-19 vaccination’s impact on case numbers, hospitalisations and deaths in people aged 80 years and older, 15 EU/EEA countries, December 2020 to May 2021
Source: Euro Surveill. 2021 Dec 2;26(48):2101030. doi: 10.2807/1560-7917.ES.2021.26.48.2101030 (PMC8641072; doi:10.2807/1560-7917.ES.2021.26.48.2101030)
Supplement: Supplementary Material [file 21-01030_NICOLAY_Supplementary_Material.pdf]

## Supplementary material

This supplementary material is hosted by *Eurosurveillance* as supporting information alongside the article "Initial assessment of the COVID-19 vaccination's impact on case numbers, hospitalisations and deaths in people aged 80 years and older, 15 EU/EEA countries, December 2020 to May 2021" on behalf of the authors who remain responsible for the accuracy and appropriateness of the content. The same standards for ethics, copyright, attributions and permissions as for the article apply. Supplements are not edited by Eurosurveillance and the journal is not responsible for the maintenance of any links or email addresses provided therein.

### Supplementary table 1. Estimated adjusted incidence rate ratio (80+/25-59-year-old) for case notification, week 48/2020 to week 20/2021#

| | At least one dose | | Full vaccination | |
|---|---|---|---|---|
| | IRR* | (95% CI#) | IRR* | (95% CI#) |
| Vaccine uptake | | | | |
| <20% | 1 (ref.) | | 1 (ref.) | |
| 20-39% | 0.65 | (0.41-1.01) | 0.56 | (0.34-0.93) |
| 40-59% | 0.48 | (0.29-0.80) | 0.44 | (0.27-0.74) |
| 60-79% | 0.45 | (0.26-0.75) | 0.40 | (0.20-0.78) |
| ≥80% | 0.34 | (0.19-0.58) | 0.35 | (0.13-0.99) |

* Incidence Rate Ratios from Poisson regression; 95% CI: 95% Confidence interval #15 countries included: Austria, Cyprus, Czechia, Estonia, Finland, Greece, Ireland, Italy, Latvia, Lithuania, Malta, Portugal, Slovenia, Spain, Sweden

### Supplementary table 2. Estimated adjusted incidence rate ratio (80+/25-59-year-old) for hospitalisation notifications, week 48/2020 to week 20/2021#

| | At least one dose | | Full vaccination | |
|---|---|---|---|---|
| | IRR* | (95% CI#) | IRR* | (95% CI#) |
| Vaccine uptake | | | | |
| <20% | 1 (ref.) | | 1 (ref.) | |
| 20-39% | 0.59 | (0.50-0.70) | 0.54 | (0.45-0.65) |
| 40-59% | 0.53 | (0.44-0.64) | 0.47 | (0.40-0.55) |
| 60-79% | 0.48 | (0.42-0.55) | 0.35 | (0.28-0.43) |
| ≥80% | 0.30 | (0.25-0.36) | 0.22 | (0.13-0.37) |

* Incidence Rate Ratios from Poisson regression; 95% CI: 95% Confidence interval; #9 countries included: Austria, Cyprus, Czechia, Finland, Ireland, Italy, Malta, Portugal, Sweden

### Supplementary table 3. Estimated adjusted incidence rate ratio (80+/25-59-year-old) for hospitalisation notifications, week 48/2020 to week 20/2021#

| | At least one dose | | Full vaccination | |
|---|---|---|---|---|
| | IRR* | (95% CI#) | IRR* | (95% CI#) |
| Vaccine uptake | | | | |
| <20% | 1 (ref.) | | 1 (ref.) | |
| 20-39% | 0.59 | (0.49-0.72) | 0.58 | (0.47-0.71) |
| 40-59% | 0.54 | (0.44-0.66) | 0.52 | (0.44-0.61) |

| | | | | |
|---|---|---|---|---|
| 60-79% | 0.48 | (0.42-0.56) | 0.37 | (0.30-0.47) |
| ≥80% | 0.34 | (0.28-0.42) | 0.22 | (0.12-0.42) |

* Incidence Rate Ratios from Poisson regression; 95% CI: 95% Confidence interval; #7 countries included: Austria, Cyprus, Czechia, Finland, Ireland, Italy, Malta and 2 countries excluded: Portugal and Sweden

**Supplementary table 4. Estimated adjusted incidence rate ratio (80+/25-59-year-old) for hospitalisation notifications, week 48/2020 to week 20/2021#**

| | At least one dose | | Full vaccination | |
|---|---|---|---|---|
| | IRR* | (95% CI#) | IRR* | (95% CI#) |
| Vaccine uptake | | | | |
| <20% | 1 (ref.) | | 1 (ref.) | |
| 20-39% | 0.61 | (0.50-0.75) | 0.59 | (0.48-0.73) |
| 40-59% | 0.56 | (0.45-0.70) | 0.54 | (0.45-0.64) |
| 60-79% | 0.52 | (0.44-0.61) | 0.37 | (0.28-0.49) |
| ≥80% | 0.35 | (0.28-0.43) | 0.22 | (0.12-0.42) |

* Incidence Rate Ratios from Poisson regression; 95% CI: 95% Confidence interval; #6 countries included: Cyprus, Czechia, Finland, Ireland, Italy, Malta, and 3 countries excluded: Austria, Portugal and Sweden

**Supplementary table 5. Estimated adjusted incidence rate ratio (80+/25-59-year-old) for death notifications, week 48/2020 to week 20/2021#**

| | At least one dose | | Full vaccination | |
|---|---|---|---|---|
| | IRR* | (95% CI#) | IRR* | (95% CI#) |
| Vaccine uptake | | | | |
| <20% | 1 (ref.) | | 1 (ref.) | |
| 20-39% | 0.81 | (0.78-0.84) | 0.53 | (0.51-0.55) |
| 40-59% | 0.48 | (0.46-0.51) | 0.36 | (0.34-0.38) |
| 60-79% | 0.38 | (0.36-0.40) | 0.26 | (0.24-0.28) |
| ≥80% | 0.27 | (0.25-0.28) | 0.16 | (0.13-0.20) |

* Incidence Rate Ratios from Poisson regression; 95% CI: 95% Confidence interval; # 15 countries included: Austria, Cyprus, Czechia, Estonia, Finland, Greece, Ireland, Italy, Latvia, Lithuania, Malta, Portugal, Slovenia, Spain, Sweden
